# From One Technologist to Another—COVID-19 Questions Answered

**DOI:** 10.2967/jnmt.120.247866

**Published:** 2020-06

**Authors:** Dmitry D. Beyder, Mark H. Crosthwaite, James Crowley, Lyndsi M. Hay, Kimberly Kerrylin Jackson, Nancy McDonald, Lynne T. Roy, Tricia Peters, Nikki Wenzel-Lamb

**Affiliations:** 1 Barnes-Jewish Hospital, St. Louis, Missouri; 2 Virginia Commonwealth University, Richmond, Virginia; 3 Carilion Clinic, Roanoke, Virginia; 4 Northwestern Memorial Hospital, Chicago, Illinois; 5 Biomedical and Engineering Imaging Institute—Mount Sinai Hospital, New York, New York; 6 Northwestern Memorial Hospital, Chicago, Illinois; 7 Cedars Sinai Medical Center, Los Angeles, California; 8 Ridley-Tree Cancer Center, Santa Barbara, California; and; 9 Society of Nuclear Medicine and Molecular Imaging, Reston, Virginia

The COVID-19 crisis has forced Nuclear Medicine and Molecular Imaging Departments to cancel appointments, change procedures and protocols, and adapt to significant changes rapidly. Nuclear Medicine Technologists are often on the frontlines, working and communicating directly with their patients. To provide advice and guidance to the community, 8 Nuclear Medicine Technologists from around the country were interviewed—a summary of their responses is documented below.

**What protocols are Nuclear Medicine Technologists following regarding staffing the department during this crisis?**

Nuclear Medicine procedures have decreased exponentially during the COVID-19 crisis. Departments across the country are taking active measures to ensure the health and safety of their patients and nuclear medicine professionals working in the department. Staffing protocols and schedules are being revised to fit the needs and safety requirements of departments. Currently, in many institutions patients and staff are being screened on entry into the department. If a patient is deemed a COVID-19–positive patient, the Nuclear Medicine Physician is immediately contacted, and the patient is masked. In addition, it is recommended that hospital personnel treat all patients as if they are COVID-19–positive and wear appropriate personal protective equipment (PPE).

In some instances, institutions are following normal low census protocols, with other institutions developing shifts, or A and B Teams, which have technologists working slightly longer shifts during the day for a week (7–10 d), followed by a week off. This schedule allows for technologists on the various teams to not directly interact with one another. This strategy minimizes COVID-19 exposure and allows technologists time to rest and decompress, away from the hospital setting. The week off also helps to provide time for symptoms to develop, should the technologist be exposed. In addition, institutions have also initiated labor pools to assist in filling necessary shifts throughout the hospital, including necessary roles (screening, wiping down surfaces, isolation gown set-ups, labs, etc.) and using cross-trained technologists to assist in other higher volume and essential areas, such as CT.

**Technologists are worried about direct exposure to COVID-19. What advice should technologists consider to reduce concern?**

The most important way to significantly decrease exposure to the COVID-19 virus is to use proper hand hygiene (wash/sanitize hands) and to use PPE correctly. Some institutions have mandated PPE refresher courses for frontline and secondary staffing. For more information on PPE guidelines, please visit www.cdc.gov.

While the concern about access to PPE was extremely high in early March, institutions reported that they have enough PPE to get them through the crisis period, based on modeling reports. Due to early shortages, institutions have encouraged the reuse of isolation and facemasks and the reuse of eye protection. In these cases, nuclear medicine professionals are having to be trained on how to safely remove facemasks (to eat, to scratch face) and reapply to ensure no high impact areas (nose, mouth, eyes) are touched and exposure is limited. Technologists should be encouraged to shower not only before work, but also immediately on returning home. In addition, technologists should change out of all scrubs, clothing, and shoes that were worn during their shift at their front door (to not have unnecessary exposure throughout their home). Because Nuclear Medicine Technologists practice ALARA principles—time, distance, and shielding—these skills and protocols have served well to limit potential exposure of the virus.

Proper sterilization of equipment continues to be important. All rooms should be wiped down in between patients, with expanded cleaning of the imaging table or injection chair to include door handles, gantry controllers, keyboards, phones, and so on. There are a variety of cleaning solutions that will kill viruses. VIREX and Sani Wipes are 2 examples. Technologists should review cleaning instructions for their specific equipment or contact the equipment manufacturer for additional guidance. Institutions should also consider an “air” clean for rooms that have had COVID-19–positive patients.

In addition, during this time, it is important that everyone works as a team, across departments and throughout the institution. This is a time of crisis and it is ok to be worried, to have concerns, but as health-care professionals, it is the technologists’ job to maintain a level of service and compassion to all that are encountered. While some are still working in the same position that they had before this crisis, some are not, and all are under a different type of stress at home and at work. It is important that technologists take time to ensure that colleagues are ok and check on each other throughout the day.

**How should staff technologists prepare for a situation where the lead or chief technologist becomes ill with COVID-19?**

Now more than ever, departments should have contingency plans in place. While the day-to-day is somewhat unknown, having a strong crossed-trained department will ensure that when the unexpected happens the department is prepared. The department protocols that are in place should not change if the Lead Technologist falls ill. Staff management and other administrative duties will have to be mitigated and addressed by other technologists in the department. Technologists will need to ensure constant communication with one another while demonstrating patience and understanding as roles and responsibilities will be shifting. Technologists should lean on leadership within their department, whether that is the Nuclear Medicine Physician or Radiologist. If a technologist is not familiar with the administrative processes in their department or how staffing is managed, they should become familiar with them. Should the lead technologist, or any member of the health-care team, become infected with COVID-19, it is important that all team members closely monitor their temperature and symptoms—as work-related exposure could occur.

**What should a Nuclear Medicine Technologist do if they are asked to transition to another department or be furloughed? Do they have any options? What should they do if they are sent home (laid-off or furloughed)?**

Interviewed technologists reported 6 different scenarios regarding hours worked and pay received during the crisis: (1) furloughed; (2) laid-off; (3) reassigned; (4) reduced hours with reduced pay; (5) full or partial pay from employers via sick, vacation leave, short-term disability, or “crisis” pay; (6) same hours, same pay, same or amended schedule.

While most technologists hope to fall into the category of still receiving a paycheck with the same pay, for some that is not an option. As a health-care professional and Nuclear Medicine Technologist, there may be times where caring for a patient puts their own health at risk. Before COVID-19, this was not common; however, times have changed, and some essential nuclear medicine procedures do put technologists at risk to direct exposure. Technologists must weigh individual options and make the best decision for their unique situation while still maintaining their professional responsibilities as a health-care professional. While a technologist may have been reassigned to a different job to help with the crisis, they should be encouraged to help the institution by doing what is required and help wherever needed. This could be in the form of checking patients at in-take, wiping down equipment, distributing PPE, and the like.

If a technologist is furloughed or laid-off, they should use this time to their benefit. Spend quality time with their immediate family. Make sure they have all the continuing education (CE) credits needed for the year. Spend time networking with their colleagues and joining virtual meetings. Technologists should investigate what options are available to them in their state specific to unemployment. Technologists should also check with their institution’s Human Resources Department for any options that may be available to them. Monitor the stimulus packages that are being vetted through the federal government for any opportunities available to furloughed or unemployed health-care professionals. Research what grant and scholarship opportunities exist through professional associations (Society of Nuclear Medicine and Molecular Imaging [SNMMI] and others). Create or update resumes. Finally, technologists should make sure to stay in touch with their employer. This crisis will come to an end and when that time comes, technologists need to be ready to help with department ramp-up—they will need to be rested, healthy, and ready to conquer the backlog of patients waiting to reschedule procedures and the litany of new patients as a result of COVID-19.

**What are suggested guidelines that institutions should be following?**

All hospitals and institutions should be following the Centers for Disease Control (CDC) Guidelines for PPE (https://www.cdc.gov/coronavirus/2019-ncov/hcp/using-ppe.html). When scanning a suspected or confirmed COVID-19 patient, it is recommended that Nuclear Medicine Technologists wear a facemask (that covers their nose and mouth) and face shield or goggles to cover their eyes, gown, and gloves. It is essential that proper removal, disposal, or sterilization procedures are followed. In addition, universal masking—ensuring all individuals within the hospital wear masks—has been effective against the spread of COVID-19 droplets.

Hospitals should also have a point person who is monitoring the daily use and inventory of PPE. All updates regarding the usage of PPE should be communicated promptly. Interdepartmental communication as well as cross-department communication is important as patients are transferred from one imaging area to another. All rooms should be wiped down in between patients, with expanded cleaning of the imaging table or injection chair to include door handles, gantry controllers, keyboards, phones, and the like. *(Refer to equipment cleaning procedures provided by the manufacturer to ensure proper cleaning.)*

**Are there any additional guidelines that Technologists should consider implementing to keep everyone safe and healthy?**

All meetings should be conducted virtually when possible. This includes telemedicine appointments for patients. Chairs should be separated in the waiting room to ensure compliance with federal distancing guidelines. In addition, all magazines, reading materials, toys, and the like should be removed from waiting areas and examination rooms. Departments should also consider preregistration by phone for all patients, including screening for symptoms.

**What should technologists consider as “essential” procedures or treatments?**

Essential procedures or treatments are best determined based on the patient needs and conditions, referring physician requests, and internal management—these are all dependent on the institution and department and therefore vary from hospital to hospital. In most cases, any imaging that is needed for a patient to continue with treatment (oncology), cardiac symptoms, or acute immediate pain are essential procedures. In general, technologists can determine if a procedure is essential by asking the following question “is the patient’s life going to be negatively affected by delaying the procedure/treatment?” If the answer is yes, then the procedure is essential. If the answer is no, then it is important to determine what delay time frame is appropriate and will still not negatively affect the patient’s life.

**Are there procedures that technologists should consider adjusting to ensure their safety?**

Whenever possible, procedure times should be reduced and altered to ensure the safety of the patient and technologist. Examples include: (1) eliminate the ventilation portion of the V/Q study and (2) avoid stressing patients on the treadmill and opt for pharmacologic stressing whenever possible. (For more information on procedure changes, please visit www.snmmi.org/COVID-19.) In addition, if available, a portable nuclear medicine scanner should be used whenever imaging a COVID-19 patient, especially one on a ventilator, to avoid transportation of the patient to different areas within the hospital. For longer procedures, if there is an opportunity to monitor the procedure through a window or behind a shielding device, that option should be implemented.

**What should technologists tell patients who are declining procedures or treatments at this time due to the crisis?**

Interviewed technologists reported 2 types of patients: (1) those who cancel and (2) those who are asking for guidance. When a patient contacts the department regarding a procedure, it is important for the technologist to answer the questions to the best of their ability and within the Nuclear Medicine Technologist scope of practice. If a technologist is unsure of how to respond to a patient’s question or believes that it is outside the scope of practice, they should refer the patient to the Nuclear Medicine Physician or Radiologist and have the physician follow up with the patient directly to answer additional questions. The technologist should explain the risk versus benefit to the patient, specific to the procedure or treatment they are delaying.

It is important that technologists provide the options available to the patient. Should the patient wish to cancel the procedure or treatment, notify the Nuclear Medicine Physician or Radiologist so they can review the patient’s file and determine next steps. As many physicians are using telemedicine, the patient should be encouraged to also reach out to their referring physician to discuss their options and risk assessment.

**Are there resources that technologists can share with patients to help them understand the importance of their procedure/treatment despite the crisis?**

The SNMMI has a Patient Resource Center (www.snmmi.org/patientcovid19), which provides information specific to nuclear medicine and molecular imaging procedures for patients. In addition, the patient should be referred to their referring physician to discuss the importance of their procedure and determine whether cancelling is an option. If a patient must move forward with a scheduled procedure, it is important to make the patient feel comfortable and reassured that every process has been put into place to ensure the health and safety of the patient and health professional staff.

**What are some suggestions on how technologists can be sure the department is ready to go once this crisis passes?**

It is anticipated that most hospitals will have an incremental “opening” period whereby certain departments and procedures are slowly reopened with a new or expanded schedule. It is important that the Nuclear Medicine Department is ready. Technologists are encouraged to start planning meetings and to have conversations with the attending physician as well as the referring physicians. Review cancelled appointments and prioritize rescheduled appointments to the most urgent cases. Read the information provided by the hospital regarding projected COVID-19 surges and reopen plans. Make sure that there is enough PPE in inventory and begin to identify how the department will adapt to the ongoing requirements of physical distancing. Departments may have to alter their schedule and should consider extending hours or offering weekend appointments to get caught up on procedures.

**What should Nuclear Medicine Departments do to be more prepared should another crisis arise?**

Institutions learned a lot about preparation from the Ebola crisis. However, the COVID-19 crisis has brought to the forefront numerous other strategies and preparation tactics. Information and guidelines change almost daily, sometimes hourly—departments must have a strong communication plan that everyone can understand. Every hospital has had to enact crisis management protocols and have adjusted patient flow to be able to assess and treat COVID-19 patients. Some have been able to maintain close to normal operations while others have converted completely to COVID-19–only operations.

It is important to know that each crisis (i.e., natural disaster, terrorist, or pandemic) will bring a different set of needs and concerns. Plans should be distributed to all staff and communicated through training courses to ensure understanding. Institutions and departments should be encouraged to hold periodic “crisis initiation” drills whereby they practice how to implement the crisis plans should another situation arise.

As the crisis slows, institutions will begin to share their stories, good and bad, about how they managed through this pandemic. Technologists should be encouraged to read journal articles related to best practices coming from the COVID-19 crisis and work to implement these into their department procedures.

It is anticipated that there will be a “second wave” of COVID-19 and that the medical community will never be the same as it was before. As the medical community works to determine what the future will look like and how infectious diseases will play a role in everyday health care, it is important for the Nuclear Medicine Department, and the Nuclear Medicine Technologist specifically, to be a part of the discussions and solution identification.

**Are there any other concerns technologists have that have not already been discussed?**

During times of crisis, when everyone is being asked to do things they have not done, are on different schedules, and have additional responsibilities (both at home and at work), there is always a concern for mental health and wellness. Technologists should be encouraged to use the resources provided by their institution, Employee Assistance Program (EAP), Social Work, and Spiritual Care.

Technologists should be reminded to monitor their own health. Stay hydrated, get plenty of sleep, and try to exercise on a regular basis. Find an outlet for mental health such as walking, gardening, or reading. If a technologist feels ill, they should be encouraged to stay home, do not take any chances—by staying home, they are doing their part to protect their patients and their colleagues.

SNMMI members are encouraged to join the SNMMI Connect COVID-19 e-community and share their concerns and challenges with their peers. This discussion board helps to not only identify resources, but also answer questions and provide guidance on difficult issues.

